# Selected In-Season Nutritional Strategies to Enhance Recovery for Team Sport Athletes: A Practical Overview

**DOI:** 10.1007/s40279-017-0759-2

**Published:** 2017-07-12

**Authors:** Lisa E. Heaton, Jon K. Davis, Eric S. Rawson, Ryan P. Nuccio, Oliver C. Witard, Kimberly W. Stein, Keith Baar, James M. Carter, Lindsay B. Baker

**Affiliations:** 1 Gatorade Sports Science Institute, 617 West Main St., Barrington, IL 60010 USA; 2 Department of Health, Nutrition, and Exercise Science, Messiah College, Mechanicsburg, PA 17055 USA; 3 Physiology, Exercise and Nutrition Research Group, Faculty of Health Sciences and Sport, University of Stirling, Stirling, UK; 4 Functional Molecular Biology Lab, Department of Neurobiology, Physiology, and Behavior, University of California Davis, Davis, CA 95616 USA

## Abstract

Team sport athletes face a variety of nutritional challenges related to recovery during the competitive season. The purpose of this article is to review nutrition strategies related to muscle regeneration, glycogen restoration, fatigue, physical and immune health, and preparation for subsequent training bouts and competitions. Given the limited opportunities to recover between training bouts and games throughout the competitive season, athletes must be deliberate in their recovery strategy. Foundational components of recovery related to protein, carbohydrates, and fluid have been extensively reviewed and accepted. Micronutrients and supplements that may be efficacious for promoting recovery include vitamin D, omega-3 polyunsaturated fatty acids, creatine, collagen/vitamin C, and antioxidants. Curcumin and bromelain may also provide a recovery benefit during the competitive season but future research is warranted prior to incorporating supplemental dosages into the athlete’s diet. Air travel poses nutritional challenges related to nutrient timing and quality. Incorporating strategies to consume efficacious micronutrients and ingredients is necessary to support athlete recovery in season.

## Key Points

**Table:** 

Emerging evidence suggests vitamin D, omega-3 polyunsaturated fatty acids, creatine, and collagen/vitamin C are potentially beneficial micronutrients and supplements to help support recovery during the competitive season.
Future research is warranted with curcumin and, bromelain, although incorporation of naturally occurring sources of these nutrients has no known risks.
An emphasis on a well-balanced diet with the inclusion of fruits and vegetables to obtain a variety of antioxidants may be more appropriate than supplementing with individual antioxidants, as whole foods contain a more balanced profile of antioxidants compared with supplemental forms. Future research should explore antioxidant-rich fruits (e.g., tart cherry, pomegranate, and blackcurrant) because early evidence indicates a potential role in supporting recovery.
There is limited evidence to support specific nutritional recommendations to reduce symptoms of jet lag with air travel. Following personalized nutrition recommendations for macronutrients and fluids to promote recovery after competition is recommended during air travel.

## Introduction

In-season recovery involves a systematic approach for maintaining athletes’ physical and mental readiness to perform in the next competition. Multiple variables can influence recovery, such as nutrition [1–3], sleep [4–7], and travel [4, 8]. In many team sports, there is limited opportunity to recover owing to high training/competition loads [9] and vigorous travel schedules (e.g., baseball, basketball, ice hockey, and soccer), which can involve inconsistent sleep schedules, time zone changes [8], and changes in altitude [10]. In addition to these issues, team sport training/competition presents significant mechanical loads (particularly contact sports, e.g., American football, rugby, and ice hockey) and metabolic demands that cause fatigue and pose challenges to the recovery process. Upon completion of the training bout or competition, the athlete enters a recovery phase in which their body restores fuel levels (metabolic recovery) and repairs damage to the musculoskeletal system (mechanical recovery).

Numerous factors can enhance recovery; chief among these are nutrition and rest. Nutrition promotes muscle regeneration [11], glycogen restoration [12], reduces fatigue, and supports physical and immune health, which helps the athlete prepare for the next competition or training session throughout the duration of a season. Nutritional aspects of recovery have primarily focused on the macronutrients, carbohydrates, and protein, as well as fluids [1–3]. Other nutritional strategies to promote recovery through the use of micronutrients and/or supplements, as well as nutritional recommendations around travel have often been considered in isolation [6, 13, 14] but rarely presented collectively.

There is a need to understand additional nutritional strategies that athletes can implement to enhance recovery and readiness throughout the season. The intent of this review is to provide (1) a brief overview of the foundational aspects of macronutrients for recovery; and (2) a summary of specific micronutrients, supplements, and nutritional strategies during recovery and travel for team sport athletes. It is not the aim of this review to provide an exhaustive list of micronutrients and supplements used to enhance recovery, nor is the intent to disassociate recovery-based nutrition strategies from those that may enhance training adaptation. Rather, we discuss selected micronutrients and supplements that are popular dietary strategies among athletes, along with emerging and novel research on their efficacy to enhance recovery during the competitive season. Specifically, this article reviews omega-3 polyunsaturated fatty acids (n-3 PUFA), vitamin D, antioxidants, creatine, curcumin, bromelain, gelatin/collagen, and vitamin C. The term ‘recovery’ is used throughout the article to describe in-season recovery strategies for team sport athletes on muscle repair and remodeling, immune function, and mediating inflammation. We present practical recommendations and applications for implementation during the season based on the available evidence to date.

## Overview of Protein, Carbohydrates, and Fluids

### Protein

At the macronutrient level, optimal dietary protein intake provides a foundational aspect for promoting recovery of team sport athletes. The multifactorial role for protein in recovery includes facilitating muscle repair, muscle remodeling, and immune function. The intermittent and multi-directional nature of movement patterns performed by team sport athletes [15, 16] require repeated eccentric muscle contractions and explain, at least in part, the indices of muscle damage (e.g., soreness and impaired function) often experienced 2–3 days after match-play [17–19]. In the context of repairing damaged muscle protein, mixed results have been reported in studies administering intact protein sources [20–23]. By contrast, several studies have replicated the finding that branched chain amino acid ingestion [24–26] or milk consumption [27–29] accelerates recovery from muscle damaging exercise.

Coupled with the repair of old damaged muscle proteins, remodeling new functional muscle proteins is also important for promoting recovery of team sport athletes. A key component of the muscle remodeling process is muscle protein synthesis (MPS); the synthesis of amino acids into functional contractile myofibrillar proteins and energy producing mitochondrial proteins. Multiple factors, including the source, per meal dose, daytime pattern and timing (in relation to exercise) of ingested protein, as well as co-ingestion of other nutrients, all modulate the response of MPS to protein intake [30]. Leucine-rich rapidly digested sources, such as whey protein, have been shown to elicit a greater stimulation of MPS during recovery compared with slowly digested proteins of lower leucine composition, such as soy, micellar casein [31], and wheat [32]. Leucine increases MPS by directly activating the mechanistic target of rapamycin complex 1 (mTORC1) through the leucine-binding protein sestrin2 [33]. The optimal short-term dose of protein to maximize stimulation of MPS equates to 0.25 g/kg body mass [34], except after whole body training when a greater dose (0.4 g/kg body mass) may be needed [35]. There is evidence that this optimal protein dose should be distributed evenly (e.g., four to five times) throughout the day [36]. The co-ingestion of other nutrients, such as carbohydrates [37–39] or n-3 PUFA [40] (as highlighted in Sect. 3) with protein confers no advantage in terms of muscle remodeling when a recommended protein dose is consumed. By contrast, the finding that drinking a large quantity of alcohol (1.5 g/kg, ~12 standard drinks) impaired the post-exercise MPS response to protein ingestion [41, 42] through the inactivation of mTORC1 [43] implicates a detrimental role for binge alcohol consumption during the muscle remodeling process.

As a closing remark, the importance of dietary protein in promoting recovery of team sport athletes may extend beyond facilitating the repair and remodeling of skeletal muscle proteins. Indeed, there is preliminary evidence that increasing dietary protein intake enhances immune surveillance during intensified training in trained cyclists [44]. These data may have important implications for teamsport athletes during periods of intense training (i.e., pre-season) and/or competition. See Table 1 for practical strategies related to sources and dosages of protein.

**Table 1:** Micronutrients and supplements dosage, sources, and benefits

Nutrient	Dosage	Best sources	Benefits	Strength of evidence^a^
Protein	0.3 g/kg as soon as possible post-training	Leucine-rich complete proteins: whey and milk	Support muscle protein synthesis Support muscle repair and muscle remodeling	Good
	0.3 g/kg/meal across 4–5 meals	Complete proteins: lean meats, poultry, fish, eggs, milk, yogurt, soy, tofu, quinoa		Good
Carbohydrate	1–1.2 g/kg within the first hour post-training	Quickly digested and absorbed: sports drinks, bars, shakes, white bread	Replenish glycogen Support immune function Reduce risk of overtraining	Good
	5–7 g/kg/day spread throughout the day	Whole grains, potatoes, sweet potatoes, brown or wild rice, fruits, vegetables, dairy products		Good
Fluid	1.0–1.5 L of fluid for each 1-kg body mass lost	Chilled fluid with sodium (20–50 mmol/L)	Restore body fluid balance and plasma volume	Good
Creatine monohydrate	20 g/day for 5 days followed by 3–5 g/day to increase and maintain elevated muscle creatine Or 3–5 g/day for about 30 days to increase muscle creatine	Meat, poultry, fish	Support training adaptations via increased expression of growth factors, reduced inflammation, and enhanced glycogen re-synthesis	Good
n-3 PUFA	~3 g/day of EPA/DHA	Cold water fatty fish (tuna, salmon), fish oils, krill oil	Reduce inflammation Support immune function Support muscle repair and remodeling when protein intake is insufficient	Fair
Vitamin D	RDA (adults) 600 IU/day Vitamin D status (blood 25OHD) 20–50 ng/L	Sunlight, supplements, fortified foods, fatty fish, egg yolk	Support muscle repair and recovery	Fair
Antioxidants	Individual antioxidant supplementation is not recommended Aim to consume a balanced diet containing a variety of fruits and vegetables	Whole fruits and vegetables and 100% fruit and vegetable juices	Reduce inflammation	Fair
Gelatin/collagen + vitamin C	≥15 g of collagen hydrolysate with ≥50 mg of vitamin C delivered 1 h before training	Gelatin, vitamin C-rich foods (e.g., oranges, raspberries, grapefruit), dietary supplements	Promote collagen synthesis	Fair
Curcumin	Dose dependent on bioavailability 0.4–5 g/day	Turmeric, dietary supplements	Reduce inflammation	Limited
Bromelain	900–1000 mg/day	Pineapple, dietary supplements	Reduce inflammation	Limited

*DHA* docosahexaenoic acid, *EPA* eicosapentaenoic acid, *n-3 PUFA* omega-3 polyunsaturated fatty acids, *RDA* recommended dietary allowance, *25OHD* 25-hydroxyvitamin D

^a^Strength of evidence conclusion statements are assigned a grade by the authors based on the systematic analysis and evaluation of the supporting research evidence. Grade I = good; grade II = fair; grade III = limited; grade IV = expert opinion only; and grade V = not assignable (because there is no evidence to support or refute the conclusion). See grade definitions at http://www.andevidencelibrary.com/

### Carbohydrates

The advice for team sport athletes engaged in periods of intensified training is to consume a high-carbohydrate diet (5–7 g/kg/day; [45]), including in the hours following exercise. The rationale behind such a diet during intensified training includes the support of daily fueling demands [12], mitigation of energy deficit, fatigue, and associated injuries [46, 47], maintenance of immune function, and prevention of overtraining [10]. Inadequate endogenous carbohydrate availability is associated with impaired team sport performance [48, 49]. As such, scenarios in which multiple training sessions are scheduled for the same day (e.g., pre-season) or during periods of intense training and/or competition (e.g., tournament play) have the potential to deplete endogenous carbohydrate stores. Even after a single competitive soccer match, it can take up to 72 h for complete muscle glycogen restoration despite dietary regimes that target carbohydrate (and protein) replacement [50, 51]. In these scenarios, with the primary goal to restore depleted muscle and liver glycogen stores as quickly as possible [1], practical recovery-focused carbohydrate recommendations for team sport athletes include the consumption of 1.0–1.2 g/kg/h within the first hour of exercise cessation and the continuation of a carbohydrate intake of 1.0–1.2 g/kg/h for 4–6 h, or until regular meals resume [46].

A variety of carbohydrate sources from both food and fluids are effective in restoring glycogen stores; the choice being determined by athlete preference (e.g., taste), practicality (e.g., two-a-day sessions), and availability (e.g., post-match travel; stadium/event offerings). Moderate-to-high glycemic-index carbohydrate choices are prudent because glycogen storage will, in part, be regulated by rapid glucose supply and insulin response [52]. Sucrose may be preferential over glucose, owing to enhanced liver glycogen repletion [53] and, where the intake of carbohydrates is sub-optimal, the addition of protein (0.3–4 g/kg/h) may help maximize glycogen resynthesis [54]. Finally, alcohol should be limited post-exercise as suboptimal dietary choices that often accompany alcohol may compromise muscle glycogen replenishment [55].

Less emphasis is placed on optimizing carbohydrate guidelines for recovery in team sport athletes when exercise intensity is low to moderate, exercise duration is short (<90 min), and when ample time separates the next exercise occasion (>8 h). In such scenarios, regularly spaced and nutrient-dense meals are likely sufficient to meet the recovery demands of the athlete. Finally, a flexible, periodized, and personalized approach to carbohydrate availability during the post-exercise period is essential to ensure short-term recovery is optimized and longer term adaptation enhanced [47].

### Fluids

Rehydration (i.e., replacement of sweat losses and restoration of body fluid balance) is an important part of the recovery process, particularly when athletes need to participate in a subsequent exercise session within a short timeframe (e.g., two-a-day practices or tournament match-play) [56, 57]. Indeed, commencing exercise in a hypohydrated state can impair performance, especially when training or competing in the heat [58].

The composition of a beverage consumed after exercise can have a significant impact on the rehydration process. The presence of sodium (~20 to 50 mmol/L) enhances palatability and stimulates physiological thirst; whereas consumption of plain water reduces the drive to drink before body water volume is fully restored [59, 60]. In addition, it is clear that sodium significantly improves post-exercise rehydration through its impact on fluid retention [57]. The increase in plasma sodium concentration and osmolality with sodium ingestion stimulates renal water reabsorption (i.e., decreases diuresis) and promotes plasma volume restoration [61–63].

Carbohydrate solutions ranging from 6 to 12% have been shown to promote greater fluid retention compared with electrolyte-matched placebos [64–67]. The increased energy density and/or osmolality of highly concentrated carbohydrate solutions (e.g., 10–12%) could delay gastric emptying or intestinal absorption [64, 65, 67]. In turn, this would slow the appearance of fluid into the circulation and attenuate diuresis during rehydration. Other proposed mechanisms, particularly regarding the fluid retention benefits of lower carbohydrate concentrations (e.g., 6%), include an insulin-mediated increase in renal sodium and water reabsorption [66].

Milk, which contains a comparable amount of sodium as sports drinks (~20–30 mmol/L), has also been studied for its efficacy in promoting fluid retention during rehydration. Recent research suggests that milk protein (80% casein, 20% whey) can enhance post-exercise fluid retention compared with traditional sports drinks (i.e., 6–8% carbohydrate-electrolyte solutions) [68–72]. However, most studies report that whey protein per se does not confer improved fluid retention compared with water or sports drinks [73–76]. The mechanism to explain these results may be that clotting of the casein in milk delays gastric emptying [70] and slows intestinal fluid absorption compared with whey protein [77] or glucose drinks [78]. However, more research is needed to understand the mechanisms underlying fluid retention improvements reported with the ingestion of protein, as well as carbohydrate-containing beverages after exercise.

In summary, beverage composition is an important consideration for post-exercise rehydration and the components found to have a significant positive impact are sodium, carbohydrates, and milk protein [56, 79, 80]. To achieve rapid and complete rehydration, expert panels recommend athletes drink 1.0–1.5 L of a sodium-containing (20–50 mmol/L) fluid for each kilogram of body mass lost [56, 57, 81]. Providing a chilled beverage with flavor and sweetness can improve beverage palatability and voluntary fluid intake after exercise [81]. Drinks with more than 2% alcohol should be avoided, as the diuretic effect is likely to impair rehydration [80]. A more comprehensive discussion on beverage composition/characteristics and rehydration can be found elsewhere [79, 80].

## Omega 3 Fatty Acids

The n-3 PUFA are a group of polyunsaturated fatty acids characterized biochemically by a double bond at the third carbon from the methyl end of the carbon chain. The n-3 PUFA are essential fatty acids, meaning they must be consumed through dietary sources. Dietary and supplemental sources of n-3 PUFA include cold water fatty fish such as tuna and salmon, fish oils, and krill oil. The most bioactive of the n-3 PUFA are eicosapentaenoic acid (EPA) and docosahexaenoic acid (DHA) [82]. Recently, n-3 PUFA have received considerable attention in the context of nutritional support for recovery. This attention stems from scientific rationale underpinning a role for n-3 PUFA in promoting muscle remodeling, muscle repair, and immune surveillance. However, a limited number of studies investigating the role of n-3 PUFA in recovery have been performed in elite athletes. The critical evaluation of n-3 PUFA and recovery must rely in part on extrapolating data from studies in recreationally trained/untrained humans and animal studies.

A topic of recent interest concerns the role of n-3 PUFA in facilitating the remodeling of skeletal muscle proteins during recovery. As highlighted in Sect. 2.1, the stimulation of MPS forms a key component of the muscle remodeling process during recovery. As such, there is current interest in the synergistic role of other nutrients alongside protein for increasing the utilization of ingested protein for stimulation of MPS during recovery [30]. Proof-of-concept studies in young [83] and older [84] adults demonstrated that 8 weeks of fish oil-derived n-3 PUFA (1.86 g of EPA and 1.50 g of DHA) supplementation increased MPS, and the phosphorylation status of cell signaling proteins known to upregulate MPS (e.g., Akt/mTORC1), in response to the intravenous infusion of amino acids and insulin. The mechanism proposed to explain this priming action of n-3 PUFA in stimulating MPS involves the direct incorporation of n-3 PUFA into the muscle phospholipid membrane [83, 85]. Such structural modifications to the muscle cell membrane are associated with an increased activation of membrane-bound cell signaling proteins, including focal adhesion kinase, Akt, and mTORC1 [85]. Because experimental studies in cell culture reveal that EPA, rather than DHA, is the active ingredient stimulating MPS [82], these proof-of-principle studies suggest a role for EPA-rich n-3 PUFA in facilitating muscle remodeling.

A physiologically relevant follow-up study in resistance-trained young male individuals demonstrated that 8 weeks of fish oil supplementation failed to modulate rates of MPS in response to feeding 30 g (0.35 g/kg) of whey protein following resistance-based exercise [40]. Thus, when ingesting a protein dose known to stimulate a maximal response of MPS [86, 87], fish oil supplementation confers no advantage for skeletal muscle remodeling during recovery. Future work is warranted to investigate the influence of n-3 PUFA supplementation on the response of MPS to ingesting a suboptimal protein dose. These data may reveal a context-specific role for n-3 PUFA in facilitating skeletal muscle protein remodeling if the athlete is unable to tolerate ingesting an optimal (~0.3 g/kg) dose of protein during recovery [40]. Moreover, given that a rodent study reported an amelioration of muscle mass loss during limb immobilization with fish oil supplementation [88], preliminary evidence substantiates a rehabilitative/prehabilitative role for n-3 PUFA during catabolic situations such as injury-induced leg immobilization that are common in many team sports. As a note of caution, a potential side effect of n-3 PUFA intake is blood thinning [89]. Therefore, athletes with a history of bleeding issues should consult with a physician before taking large doses of n-3 PUFA.

The role of n-3 PUFA also has been investigated in the context of less severe soft-tissue injuries caused by intense exercise. The anti-inflammatory properties of n-3 PUFA are proposed to ameliorate feelings of muscle soreness and impairments in muscle function associated with eccentric exercise [90]. The model most commonly employed by laboratory-controlled studies to elicit eccentric exercise-induced muscle damage consists of untrained volunteers performing repeated muscle contractions using an isokinetic dynamometer. Hence, the external validity of study findings to recovery from team-based sporting activities must be considered with caution. Nevertheless, studies have shown a protective role for n-3 PUFA intake in attenuating muscle soreness [91, 92] and oxidative stress [93] 48 h after exercise. Given the direct incorporation of n-3 PUFA into the muscle cell membrane [85] and the potential for n-3 PUFA to modify the structural integrity of the cell membrane, these preliminary data suggest a protective role for n-3 PUFA in reducing the muscle-damaging effects of eccentric-based muscle loading. Future studies investigating the protective role of n-3 PUFA during short-term recovery should be conducted in high-performance athletes, simulate real-world muscle-damaging exercise (e.g., match-play), and include valid sport-specific performance measurements.

The n-3 PUFA also exhibit immunomodulatory properties. In addition to initiating anti-inflammatory mediators, termed resolvins [94], EPA and DHA also alter neutrophil proliferation and monocyte phagocytosis [95]. Two recent studies implicate a role for n-3 PUFA in improving the immune status of recreationally trained volunteers during recovery [93, 96]. Six weeks of fish oil supplementation (3 g/day, 1.3 g of EPA and 0.3 g of DHA) increased interleukin (IL)-2 production and the cytotoxic activity of natural killer cells during 3 h of exercise recovery [93]. Consistent with these short-term findings, a recent longitudinal study reported fewer symptoms of upper respiratory tract infection when volunteers received a fish-oil-containing supplement during 16 weeks of training [96]. Taken together, these preliminary results suggest a potential role of n-3 PUFA in improving immune status over the course of a season in team sport athletes and thus warrant further investigation. See Table 1 for practical strategies related to the sources and dosages of n-3 PUFA.

## Micronutrients

### Vitamin D

Vitamin D, also known as the ‘sunshine vitamin’, is classified as a steroid hormone for which receptors are present in a variety of tissues throughout the body, such as the small and large intestine, prostate, brain, pancreas, and skeletal muscles. Individuals obtain vitamin D precursors from sun exposure or diet. The amount of vitamin D obtained from sun exposure is highly variable, depending on factors such as latitude, environment, season, skin pigmentation, clothing, and sunscreen use. Therefore, obtaining vitamin D from the diet or supplements may be important to maintain appropriate status. The Institute of Medicine’s recommended dietary allowance for vitamin D is 600 IU/day for individuals aged 1–70 years [97], although some researchers claim this value is low [98]. Debate also exists on the tolerable upper intake level, set at 4000 IU/day, with symptoms of toxicity unlikely at 10,000 IU/day [97]. Because of athlete compliance, a common practice is to megadose weekly with high-dose vitamin D supplements; however, recent research suggests this is a practice that should be considered with caution and may be ineffective [99].

An aspect of recovery following intense exercise is the repair of damaged muscle tissue via satellite cell activation. While many factors influence this repair process, emerging data suggest a role for vitamin D [100]. Research in animal cell models indicates that treatment with vitamin D may play a role in muscle regeneration via satellite cell activation followed by myoblast proliferation, migration, and differentiation (see a recent review [100] for further details). Vitamin D treatment resulted in improved migration, and myotube differentiation [101] in a muscle biopsy of vitamin D-deficient subjects after mechanical injury. Research in a rodent model has demonstrated improved cell proliferation and decreased apoptosis following muscle injury (crushing) with vitamin D treatment [102]. Taken together, this work conducted in isolated muscle cells indicates a role for vitamin D in the repair dynamics of skeletal muscle.

Four studies have been published to date related to the specific role of vitamin D for muscle recovery in humans. Muscle weakness (measured as peak isometric force or peak torque) was chosen as the measure of recovery because it is reflective of both degeneration and regeneration, remains suppressed until repair is complete, and is a functional outcome for the athlete [103]. Ring et al. [104] did not find an association between baseline vitamin D status (measured as blood 25OHD level) and muscle pain or peak isometric force following eccentric exercise of the elbow flexors up to 4 days post-insult. By contrast, using lower body exercise, Barker et al. [103] found that pre-exercise vitamin D status of recreationally active individuals was significantly correlated with immediate and longer term (48 and 72 h) muscle weakness following intense exercise in an exercised leg vs. control leg.

While correlating vitamin D status to functional outcomes indicates a possible relationship, intervention studies are needed to determine whether improving status can result in improved recovery. In a follow-up study, Barker et al. [105] supplemented healthy, moderately active, adult male individuals with 4000 IU/day or placebo for 35 days. After 28 days of supplementation, subjects completed a one-leg eccentric protocol to induce muscle damage. Recovery of peak isometric force, but not muscle soreness, was significantly improved (~8%) in the supplement group from immediately post to 24 h post-exercise, but not any other time point (48, 72, or 168 h). A major limitation of this study was that vitamin D status was not accounted for at baseline and groups were not randomized based on initial status.

A carefully controlled intervention protocol conducted by Owens et al. [101] also found vitamin D supplementation to improve recovery indices. Moderately active, adult male individuals with deficient vitamin D status at baseline were supplemented with 4000 IU/day or placebo for 6 weeks. Before and after supplementation, subjects completed eccentric exercise to induce muscle damage of the knee extensors followed by peak torque measurement over 7 days of recovery. Peak torque was improved in the vitamin D-supplemented group at 48 h and 7 days post-exercise, as compared with placebo. The authors suggested these were promising preliminary data, but further studies are needed with a larger sample size and varying exercise protocols to induce muscle damage.

In summary, more work is necessary to clarify the benefit of vitamin D for athletic muscle recovery, including the interaction with protein intake. Unlike the recommendation to consume protein shortly following athletic activity, shrot-term vitamin D consumption will likely not influence muscle repair. Athletes should aim to maintain appropriate vitamin D status via regular sun exposure, supplementation, and/or diet. The target level of 25OHD has not been identified for an endpoint of muscle repair; however, it seems prudent for athletes to aim for the clinical cutoff for sufficient vitamin D status, which is 30–50 nmol/L [100]. Despite these and other outstanding questions, the available data suggest vitamin D may play a role in the muscle repair and recovery process. See Table 1 for practical strategies related to sources and dosages of vitamin D.

### Antioxidants

Exogenous antioxidants include vitamin E, vitamin C, and carotenoids [106], as well as flavanols (e.g., catechins), flavonols (e.g., quercetin) and anthocyanidins (e.g., cyanidin) [107]. Endogenous antioxidants (e.g., superoxide dismutase and glutathione peroxidase) scavenge reactive oxygen species (ROS) [107]. Both endogenous and exogenous antioxidants work in synergy to protect the body from damage caused by free radicals and maintain redox balance [107, 108]. It is important to note that excessive amounts of free radicals or antioxidants can be problematic owing to the disruption of redox balance [107].

While strenuous exercise increases oxidative stress, it also appears to upregulate endogenous antioxidant production (i.e., hormesis), resulting in beneficial effects including increased activity of antioxidant enzymes and repair of oxidative damage [108]. Research indicates that ROS are important signaling molecules for adaptations to occur in the skeletal muscle [109, 110], while low levels of ROS are needed to support muscular force production [111]. As such, large amounts of antioxidants may impair recovery by blunting the regenerative process that ROS support [112, 113].

Research regarding the effects of antioxidants on training adaptations and recovery has produced mixed results. Thompson et al. [114] showed a modest improvement in exercise recovery (e.g., lower plasma IL-6 levels 2 h after exercise, reduced muscle soreness 24–48 h after exercise) following 2 weeks of vitamin C supplementation (400 mg/day). Jakeman and Maxwell [115] provided 400 mg/day of vitamin C, 400 mg/day of vitamin E, or placebo for 21 days before and for 7 days after a 60-min eccentric exercise protocol. The vitamin C intervention improved recovery of maximal contractile function 24 h following the eccentric exercise protocol. The 400 mg/d of vitamin E had no effects on muscle contractile function [115].

Interestingly, the combination of vitamin C (1000 mg/day) and vitamin E (400 IU/day) may negatively impact certain cellular adaptations to endurance training [116]. In one study, subjects ingested vitamin C (1000 mg/day) and vitamin E (235 mg/day) for 11 weeks while participating in an endurance training program [117]. After training, increases in mitochondrial biogenesis markers (i.e., mitochondrial proteins) were attenuated in the antioxidant intervention compared with placebo [117]. Another study administered a vitamin supplement containing 400 mg of vitamin C, 268 mg of vitamin E, 2 mg of vitamin B6, 200 µg of folic acid, 5 µg of zinc sulfate monohydrate, and 1 µg of vitamin B_12_ or placebo over a period of 6 weeks [118]. There were no differences between the supplement and placebo groups in inflammation or muscle function 7 days after muscle damaging exercise [118]. Finally, antioxidants delivered as a cocktail (272 mg of α-tocopherol, 400 mg of vitamin C, 30 mg of β-carotene, 2 mg of lutein, 400 µg of selenium, 30 mg of zinc, and 600 mg of magnesium) long term over a period of 4 weeks had no impact on muscle damage or exercise-induced inflammation after subjects completed a 1000-km kayaking race [119].

The studies related to antioxidants and training adaptations/recovery raise questions about intentional supplementation. With regard to vitamin C, >1 g/day may negatively impact training adaptations while lower amounts (up to 250 mg/day) occurring naturally in fruits and vegetables may not [120, 121]. Vitamin E supplementation may be efficacious in some situations, but applications are very limited (e.g., acutely, surrounding competition at altitude) and long-term consumption is not advised [120]. Supplementation of vitamin C or vitamin E in isolation or combined show little benefit protecting against muscle damage and supplementation with large doses may negatively impact ROS signaling functions [122]. In short, some studies have demonstrated that antioxidants may blunt adaptations, while others have shown no detrimental effect on various responses to exercise (e.g., mitochondrial biogenesis). These conflicting results may be, in part, owing to the difference in baseline antioxidant status, antioxidants delivered, exercise training protocols, and/or dosing strategies used in the various studies [110]. In a review, Braakhuis and Hopkins [120] have suggested that (1) long-term supplementation with dietary antioxidants may be detrimental to training adaptations, and (2) long-term intake of certain polyphenols such as epicatechin or resveratrol may provide a benefit when paired with exercise training. However, the review also concluded additional research is warranted.

Additional studies have examined the effect of dietary sources of antioxidants, such as fruits and vegetables, on recovery. Beetroot juice, commonly ingested for its potential performance-enhancing effects [123], has also demonstrated a role in supporting training adaptations both short term [124] and long term [125]. Some emerging evidence suggests that beetroot juice may support aspects of exercise recovery by mitigating loss of muscle function [126] and soreness [126, 127] after certain types of exercise [127]. However, not all studies have demonstrated a benefit with beetroot juice ingestion on mitigating soreness or exercise-induced inflammation post-exercise [128].

There is also an emerging body of literature on the effects of other antioxidant rich fruits such as tart cherry [129, 130], pomegranate [131, 132], and blackcurrant (BC) [133] on recovery. Tart cherry has been shown to reduce markers of inflammation [134], decrease perceptions of soreness [129], and improve redox balance [134] compared with placebo after exercise. Pomegranate juice has been shown to reduce muscle soreness and weakness in elbow flexors following an eccentric elbow flexion protocol [132]. Ammar et al. [131] showed that pomegranate juice lowered the perception of knee extensor soreness and attenuated the acute rise in specific markers of tissue damage [e.g., creatine kinase (CK), lactate dehydrogenase] 3 min after Olympic weight-lifting movements, and improved the recovery kinetics of these same markers 48 h after exercise. Blackcurrant has been studied for its effects on exercise performance [135, 136], substrate oxidation [137], and physiological measures, such as blood lactate level [135, 136, 138]. Only one study has tested the effects of BC on exercise-induced muscle damage, subjective ratings of muscle soreness, and inflammation post-exercise [133]. In a parallel-design study, moderately active subjects consumed 16 oz of a BC nectar or placebo twice daily over the course of 8 days. On the fourth day, subjects completed a series of eccentric squatting exercises. The BC group had lower plasma IL-6 levels than the placebo group after 24 h but not 48 or 96 h post-exercise. At both 48 and 96 h post-exercise, CK was lower in the BC group compared with the placebo group. However, there were no differences in soreness post-exercise with BC compared with placebo. The lack of crossover design limits the interpretation of the data. Additional research is warranted to determine the efficacy of BC.

Additional research is needed to understand the impact that different antioxidants may have on recovery and training adaptations. Although not conclusive, situations where antioxidant supplementation may be advantageous are during times when recovery is paramount, such as when an athlete has multiple training bouts or competitions in a short period of time or when training/competing at altitude [120, 139]. Because of the potential for antioxidant supplementation to blunt training adaptations, caution should be used when the athlete is training to improve aerobic capacity or maximize strength gains. Consideration of antioxidant use also rests within the timing of the season and caliber of athlete. In the collegiate or professional setting, athletes may seek to increase their adaptive response to training during the off-season, while their focus may shift to maintenance during the season. High school athletes may differ in that they transition to different sports from season to season. Until more is known, focusing on a well-balanced diet including fruits and vegetables to obtain antioxidants may be a more appropriate alternative to supplementing with individual antioxidants [108]. There seems to be no evidence at this time to suggest that consumption of fruits and vegetables blunts exercise-induced adaptations [109].

## Creatine Monohydrate

Creatine is a non-essential nutrient that is produced endogenously in the liver, pancreas, and kidneys, and is also consumed through the diet [140]. The primary sources of dietary creatine are meats and fish with concentrations ranging from 3 to 5 g of creatine per kg raw meat, although some fish, such as herring may contain up to 10 g/kg. About 95% of the creatine in the human body is stored in skeletal muscle where, along with phosphorylcreatine and the enzyme CK, it is involved in adenosine triphosphate synthesis. The CK reaction is a particularly important source of adenosine triphosphate during times of high energy demand, such as maximal exercise.

Creatine monohydrate supplementation (about 20 g/day for 5 days) increases muscle creatine by ~20% [141] and subsequently improves the performance of exercises that rely heavily on creatine and phosphorylcreatine to resynthesize adenosine triphosphate [142, 143]. An overwhelming majority of the research on safety [144] and efficacy has focused on creatine monohydrate. No advantage has been shown using different formulations of creatine, which typically contain less creatine and can be more expensive [145]. In this review, unless otherwise specified, creatine supplementation will refer to creatine monohydrate supplementation.

While the pre-exercise performance-enhancing effects of creatine supplementation have been well documented, several studies also point to a potential role for creatine as a post-exercise recovery aid. A dietary supplement could prove useful as a recovery aid, if it enhanced fuel replacement, increased post-exercise MPS, enhanced growth factor expression, and/or reduced exercise-induced muscle damage and inflammation. It seems that increasing muscle creatine via creatine monohydrate supplementation supports many of these benefits [146, 147].

Following intense exercise, muscle phosphocreatine and glycogen are depleted, but creatine supplementation may enhance recovery of these important fuel sources. Greenhaff et al. [148] and Yquel et al. [149] demonstrated enhanced post-contraction/exercise phosphocreatine re-synthesis, although this has not been replicated in every study [150]. As post-exercise phosphocreatine resynthesis takes several minutes to complete, faster re-synthesis may enhance recovery from a short-term bout of exercise and thereby improve performance in a subsequent bout. Similarly, several studies have reported increased muscle glycogen following creatine supplementation (reviewed in [151]). As an example, Nelson [152] reported a 12% increase in muscle glycogen when carbohydrate loading occurred after 5 days of creatine loading. Roberts et al. [153] showed an 82% greater increase in muscle glycogen resynthesis during the first 24 h of carbohydrate plus creatine loading compared with carbohydrate loading alone. Improved post-exercise glycogen re-synthesis with creatine ingestion could enhance a subsequent bout of exercise hours or days later.

The effects of creatine supplementation on MPS and muscle protein breakdown (MPB) have been investigated under various conditions [154–156]. Parise et al. [156] found that creatine supplementation reduced plasma leucine rate of appearance and leucine oxidation rate (in men), but speculated that this was from liver or splanchnic proteins. Louis et al. [154, 155] found no effect of creatine supplementation on protein turnover in post-exercise, post-absorptive, and fed states. Thus, it seems that increased fat-free mass subsequent to creatine supplementation is not mediated directly through measureable increased MPS or decreased MPB. However, other groups have demonstrated that creatine supplementation may be valuable for recovery through the increased expression of proteins and growth factors or cells that participate in the muscle remodeling process [157–160].

Willoughby and Rosene [159, 160] reported that in addition to a greater increase in fat-free mass and muscle volume and strength, creatine supplementation (6 g/day for 12 weeks) increased myofibrillar protein, Type I, IIa, and IIx myosin heavy chain messenger RNA (mRNA) expression, type I and type IIx myosin heavy chain protein expression, CK, myogenin, and MRF-4 mRNA expression, and myogenin and MRF-4 protein expression compared with resistance training and placebo ingestion. Further, Deldicque et al. [157] showed increased insulin-like growth factor-1 and -2 mRNA in resting muscle following creatine supplementation (21 g/day for 5 days). In addition, creatine supplementation has been shown to augment the resistance training increase in satellite cell number and myonuclei concentration [158]. Safdar et al. [161] reported that creatine supplementation increased the expression of over 270 genes including those involved with osmosensing, cytoskeleton remodeling, Glut 4 translocation, glycogen and protein synthesis, satellite cell proliferation and differentiation, DNA replication and repair, mRNA processing and transcription, and cell survival. These beneficial effects are not entirely surprising, as creatine supplementation draws water into the muscle cell [162], and it is known that cellular hyper-hydration inhibits protein breakdown and RNA degradation, and stimulates glycogen [163], protein, DNA, and RNA synthesis [164, 165]. Together, these studies indicate that creatine supplementation has the potential to support recovery through various pathways/mechanisms.

Several groups have investigated the effects of creatine supplementation on markers of exercise-induced muscle damage following eccentric [166–169], resistance [170–172], endurance [173–175], and sprint exercise [176]. Cooke et al. [166] found reduced post-exercise CK and lactate dehydrogenase, and increased strength recovery in creatine-supplemented subjects following eccentric-biased resistance exercise of the lower body. Rosene et al. [169] showed improved isometric force production following a second (30 days later), but not after the first, bout of eccentric-biased resistance exercise. Following repeated bouts of resistance exercise, Veggi et al. [172] reported that creatine supplementation attenuated increases in CK and delayed-onset muscle soreness (DOMS) and decreases in range of motion. Although beneficial effects were not noted in all investigations [170, 171], it seems that creatine monohydrate may play a role in reducing the cellular disruption associated with resistance exercise. Three separate investigations have concluded that creatine supplementation (20 g/days for 5 days) attenuated the increase in various markers of muscle damage and inflammation including: CK, prostaglandin-E2, tumor necrosis factor-α (TNF-α), lactate dehydrogenase, interferon-α, and IL-1-β following an endurance exercise challenge (20-km run, half and full distance triathlon) [173–175]. Finally, Deminice [176] reported that creatine blunted the post-sprint exercise (six 35-m sprints) increase in C-reactive protein, TNF-α, and lactate dehydrogenase, even though power production increased. The available data suggest that creatine supplementation prior to an endurance or sprint exercise challenge reduces both muscle damage and inflammation. No studies have shown increased markers of muscle damage in creatine-supplemented individuals under either resting or post-exercise conditions (reviewed in [147]).

In summary, a large number of studies support the use of creatine monohydrate as a sports performance enhancer and also as an adjunct to resistance training that can increase fat-free mass, strength, and fatigue resistance. Further, several studies indicate that increasing muscle creatine content through creatine supplementation creates an intracellular environment that encourages better recovery between short-term bouts of exercise and during long-term exercise training.

## Curcumin

Curcumin is a component of the spice turmeric and is often used to reduce inflammation. Its mechanism of action may be related to the inhibition of cyclooxygenase, TNF-α, and other proinflammatory agents [177]. The effects of curcumin have been demonstrated in studies related to inflammatory conditions such as arthritis [178]. Supplementation with curcumin to reduce inflammation and/or muscle soreness is of particular interest to athletes seeking an alternative to non-steroidal anti-inflammatory medications.

Nicol et al. [179] reported that a dose of 5 g/day of curcumin reduced DOMS 24 and 48 h after high-intensity muscle-damaging exercise [179]. This study also reported a small reduction in a marker of muscle injury (i.e., CK) [179]. Similarly, McFarlin et al. [180] reported a 48% reduction in CK after subjects consumed 400 mg/day of a highly bioavailable source of curcumin for 2 days before and 4 days after a high-intensity, muscle damage-inducing protocol. McFarlin et al. [180] also reported a ~25% decrease in circulating levels of the inflammatory cytokines TNF-α and IL-8. Neither study found a reduction in serum levels of IL-6 [179, 180]. Even though positive effects of curcumin have been found during intense eccentric muscle injury protocols, endurance exercise trials have not produced significant reductions in DOMS or inflammatory markers [181]. Sciberras et al. gave subjects 500 mg/day of highly bioavailable curcumin for 3 days and an additional 500-mg dose immediately prior to 2 h of endurance exercise. There were no significant differences in serum IL-6, IL-1 receptor antagonist, IL-10, cortisol, or C-reactive protein post-exercise between the curcumin supplementation group, placebo, or control (no supplementation) [181].

In summary, supplementation with curcumin may be beneficial for athletes participating in high-intensity exercise with a significant eccentric load. Consuming 400 mg or more of curcumin via the spice turmeric in the diet in an effort to decrease inflammatory cytokines or reduce DOMs is unrealistic. However, highly bioavailable alternatives have been produced and may prove more useful in decreasing inflammatory issues but need to be explored further.

## Bromelain

Bromelain is a proteolytic enzyme found in both the stem and fruit of pineapple [177, 182] and has been studied as a treatment for a number of inflammatory conditions in humans [183]. The proposed mechanism of action of bromelain is reducing the production of proinflammatory prostaglandin production without affecting anti-inflammatory prostaglandins [184]. However, it is important to recognize that the primary effect of bromelain is as a protease; an enzyme that cuts other proteins and regulates clot formation and resorption after an injury [185]. Therefore, if exercise does not induce a significant membrane injury that results in fibrin clot formation, the effectiveness of bromelain may be limited.

Bromelain has been extensively studied in inflammatory disease states in the general population. There is less information available on its effects in an athletic population. It was first suggested to provide a benefit for muscular injuries in an experiment using hamsters performing eccentric exercise [182]. However, Stone et al. found that in humans, neither 1200 mg of ibuprofen nor 900 mg of bromelain was better than placebo at reducing DOMS after resistance exercise in untrained subjects [186]. Similarly, Shing et al. [187] found that even though 1000 mg/day of bromelain decreased perceived fatigue on the fourth day of a 6-day stage race, it had no effect on indices of muscle injury.

Although bromelain in isolation may have a limited effect on muscle injury in athletes, there may be a benefit when used in combination with other protease inhibitors. Buford et al. [188] showed that taking a proteolytic supplement containing bromelain, fungal proteases, and papain for 21 days improved muscle function after a downhill running protocol (−17.5% grade for 45 min). Similarly, bromelain (50 mg) in conjunction with other proteases (325 mg of pancreatic enzymes, 75 mg of trypsin, 50 mg of papain, 10 mg of amylase, 10 mg of lipase, 10 mg of lysozyme, 2 mg of chymotrypsin) taken four times a day, 1 day before and 3 days after downhill running improved muscle function 24 and 48 h after exercise when compared with placebo [189]. The particular blend of proteases used by Miller et al. [189] may improve muscle function after exercise involving a high eccentric load. However, more research is needed to understand the potential effects of proteases on DOMS in athletes as well as the underlying physiological mechanisms.

## Gelatin/Collagen and Vitamin C

Collagen is the primary structural protein in connective tissues such as bone, tendon, ligament, and cartilage. The idea of supplementing with gelatin/collagen and vitamin C is that it will provide the amino acid building blocks as well as the essential co-factor to promote greater collagen synthesis. Gelatin is a food product used to produce gummy sweets that is produced by partial hydrolysis of the collagen extracted from the skin, bones, and connective tissues of animals. Hydrolyzed collagen is further broken down so that it is soluble in water and no longer forms a gel.

The notion that gelatin and vitamin C can improve collagen synthesis in connective tissues has been confirmed using an in-vitro model of a ligament where treating with pro-collagen amino acids and vitamin C increased collagen production three fold [190]. In humans, consuming gelatin 1 h before a short period of mechanical loading is able to double the amount of the amino-terminal propeptide (Procollagen I N-terminal Propeptide) of type I collagen in the blood [191].This indicates that gelatin can improve the collagen synthesis response to loading. Longer term supplementation with collagen hydrolysate has further been shown to improve cartilage function in patients with osteoarthritis [192]. In this study, McAlindon et al. [192] showed that consuming 10 g of collagen hydrolysate per day resulted in an increase in cartilage thickness in the knee. In agreement with this finding, a 24-week randomized clinical trial in athletes showed that 10 g of collagen hydrolysate significantly decreased knee pain [193]. Mouse studies using C14-labeled gelatin hydrolysate [194] demonstrated that >95% of the gelatin is absorbed after feeding. More interestingly, even though the pure amino acid proline could be incorporated into skin collagen as well as gelatin, gelatin was incorporated into the collagen of cartilage and muscle twice as much as tracer from proline [194]. These data suggest that musculoskeletal collagen synthesis is greater in response to gelatin or hydrolyzed collagen than to the individual amino acids.

Even though there are strong data to suggest that supplementing with gelatin and vitamin C can benefit connective tissues, additional research is warranted to explore the benefit to athletes. Future research is needed to determine the dose and frequency of gelatin and vitamin C ingestion needed. Additional questions include: (1) Does supplementation decrease injuries or accelerate the return to play after injury? (2) Because connective tissue plays an important role in the response to resistance exercise [195], can supplementation with gelatin and vitamin C improve performance?

## Nutritional Recommendations for Air Travel

Team sport athletes frequently travel throughout the competitive season, with some sports requiring travel immediately after competition to prepare for a game the following day. With long-distance travel, not only does the athlete face the challenge of being fatigued from competition but also from jet lag while traveling across multiple time zones. Jet lag symptoms include impaired sleep, fatigue, headaches, general malaise, and loss of concentration and motivation from the disruption of circadian rhythms [196, 197].

Nutrient timing and meal composition have been proposed as potential dietary interventions to reduce symptoms of jet lag by enhancing adaptation of circadian clocks [198–203]. Animal models support this notion showing rapidly digested carbohydrates [200] or a mixed macronutrient meal (14% protein, 72% carbohydrates, 4% fats) [198] applied in a time-restricted feeding pattern (i.e., 24-h food deprivation) can influence faster circadian rhythm adjustment to a new time zone. Amino acids and fish oils have also been shown to accelerate entrainment of the circadian clock when incorporating a jet lag model in rodents [199, 201]. Hirao et al. [198] suggested that such dietary applications could reduce symptoms of jet lag. However, the impact of these dietary interventions on athletes to modify jet lag symptoms is unclear. Moreover, the results in animal models likely have limited application to athletes because the studies employed a 24-h food deprivation protocol prior to feeding [198–201]. Interestingly, animal studies suggest that hypercaloric diets high in fat and alcohol consumption could alter circadian clock synchronization to light, resulting in a slower rate of re-entrainment (i.e., adaptation) to jet lag [204, 205].

Only two clinical trials in humans have examined meal composition as a cue to modify peripheral circadian clocks [206, 207]. Kräuchi et al. [206] showed that a carbohydrate-rich meal in the morning significantly advanced the circadian rhythm (phase advance) compared with the same meal provided in the evening. However, the meal was applied in a time-restricted feeding pattern, which may not be representative of athletes’ nutritional strategies. Over a 3-day period, subjects were only allowed to consume one carbohydrate-rich meal either in the morning or evening. In another study, Reynolds et al. [207] showed reduced jet lag symptoms in military personnel traveling from USA to South Korea when consuming the Argonne diet. Four days prior to departure, soldiers incorporated the diet, which involved alternating high-caloric days (no caloric limit) with days of low caloric intake (limited to 800 kcal) [207]. The high-calorie days consisted of high-protein meals for breakfast and lunch and carbohydrates for dinner with fruits and vegetables being consumed on low-calorie days [207].

Although the notion of incorporating nutritional strategies to reduce jet lag symptoms is attractive, currently, there is limited research to support such implementation with athletes. Instead, athletes should focus on nutritional strategies to promote recovery during air travel. Alternative methods to reduce jet lag symptoms (e.g., light exposure, melatonin) may be more effective and are reviewed elsewhere [208].

Meeting the personalized nutrition recommendations to enhance recovery as discussed in the macronutrient and fluid recommendation section (Sect. 2) presents a challenge during air travel. Factors such as limited or unfamiliar food items and lack of access to fluids are some of the challenges athletes face during commercial flights. During air travel, limited food options may not provide the adequate macronutrient content the athlete needs to recover. Further, unfamiliar food items could cause potential gastrointestinal distress [13]. It is therefore important for athletes to plan ahead when traveling for competition and to pack non-perishable food items and fluids to help meet individual macronutrient and fluid needs to enhance recovery. If traveling internationally, athletes should consider culture differences based on the location of their travel and practice proper hygiene standards to avoid potential gastrointestinal pathogens from food and water [13, 209, 210].

It has been suggested that an extra 15–20 mL of fluids should be consumed for each hour of flight owing to increased moisture losses from the respiratory tract [13]. However, this would only equate to 150–200 mL of additional fluid loss during a 10-h flight. The practical impact of incorporating additional fluids to account for respiratory losses during air travel beyond the post-exercise fluid replacement recommendations [81] is likely not warranted. Athletes should be encouraged to avoid or limit alcohol consumption post-competition during flights to promote rehydration [13, 81, 210, 211]. A more comprehensive discussion on nutrition and travel may be found elsewhere [13, 208, 210].

## Practical Applications

Many supplements and strategies exist that are claimed to support recovery and performance in-season, with varying levels of efficacy. Prior to initiating any supplementation, the athlete must consume a diet adequate in protein, carbohydrates, fat, and micronutrients. Without this foundation, the additional benefits of even efficacious supplements will be limited. The goal of a recovery meal is to provide the athlete with the nutrients needed to support MPS and glycogen repletion as well as rehydration. An 80-kg team sport athlete should aim for 20–24 g (0.25–0.3 g/kg) of protein in the recovery meal with 80–96 g (1–1.2 g/kg) of carbohydrates. This could be accomplished through the consumption of 85–113 g (3–4 oz) of meat, poultry, or fish, with 1.5 cups of pasta with marinara sauce, plus a vegetable of choice. The athlete could also include dietary sources of antioxidants and n-3 PUFA within this meal to support recovery.

Once the athlete has established this dietary foundation, supplementation with selected nutrients may provide an additional benefit to recovery, such as those listed in Table 1. In addition to focusing on evidence-based supplements, the athlete must also be aware that supplements are not well regulated and may include ingredients that are banned by the World Anti Doping Agency [212]. It is important that athletes choose supplements that have been third-party tested for quality and safety.

## Conclusions

Team sport athletes face many challenges in regard to in-season recovery. Because of the limited opportunities to recover between competitions, combined with busy travel schedules, athletes must be deliberate in their recovery strategies (Table 1). Protein, carbohydrates, and fluid are commonly acknowledged as important components of the recovery process. Specific micronutrients and supplements may also play key roles in athletic recovery related to MPS/MPB, immune function, and mediating inflammation. Emerging evidence suggests vitamin D, n-3 PUFA, creatine, and collagen/vitamin C are potentially beneficial supplements to support recovery during the season, although additional research is needed. Nutrients such as curcumin and bromelain may also have potential benefits, although further research is warranted and the dose likely to provide a benefit far exceeds what an individual could consume through food (however, inclusion of the natural sources of these nutrients would not be harmful). Future research is warranted with curcumin, and bromelain prior to incorporating supplemental doses into an athlete’s dietary recovery strategy. Consuming antioxidants via whole foods in the diet provides anti-inflammatory benefits while limiting the negative impact supplemental antioxidant intake can have on training adaptations. Special considerations must also be made to support the demands of air travel, central to which will be the need for advanced planning. Overall, a nutrient-dense diet consumed throughout the day, in combination with a few selected supplements, can support the athletes’ recovery goals during the competitive season. Finally, athletes should always seek professional advice before adopting nutritional strategies with the intent to improve recovery in-season.

## References

[1] Beelen M, Burke LM, Gibala MJ (2010). Nutritional strategies to promote postexercise recovery. Int J Sport Nutr Exerc Metab..

[2] Burke LM, Mujika I (2014). Nutrition for recovery in aquatic sports. Int J Sport Nutr Exerc Metab..

[3] Beck KL, Thomson JS, Swift RJ (2015). Role of nutrition in performance enhancement and postexercise recovery. Open Access J Sports Med..

[4] Nédélec M, Halson S, Abaidia AE (2015). Stress, sleep and recovery in elite soccer: a critical review of the literature. Sports Med..

[5] Fullagar HH, Skorski S, Duffield R (2015). Sleep and athletic performance: the effects of sleep loss on exercise performance, and physiological and cognitive responses to exercise. Sports Med..

[6] Halson SL (2014). Sleep in elite athletes and nutritional interventions to enhance sleep. Sports Med..

[7] Nédélec M, Halson S, Delecroix B (2015). Sleep hygiene and recovery strategies in elite soccer players. Sports Med..

[8] Simmons E, McGrane O, Wedmore I (2015). Jet lag modification. Curr Sports Med Rep..

[9] Halson SL (2014). Monitoring training load to understand fatigue in athletes. Sports Med..

[10] Meeusen R, Duclos M, Foster C (2013). Prevention, diagnosis and treatment of the overtraining syndrome: joint consensus statement of the European College of Sport Science and the American College of Sports Medicine. Med Sci Sports Exerc..

[11] Phillips SM, Van Loon LJ. Dietary protein for athletes: from requirements to optimum adaptation. J Sports Sci. 2011;29(Suppl. S1):S29–38. 10.1080/02640414.2011.619204 22150425

[12] Burke LM, Hawley JA, Wong SH (2011). Carbohydrates for training and competition. J Sports Sci..

[13] Reilly T, Waterhouse J, Burke LM (2007). International Association of Athletics Federations. Nutrition for travel. J Sports Sci..

[14] Todd JJ, Pourshahidi LK, McSorley EM (2015). Vitamin D: recent advances and implications for athletes. Sports Med..

[15] Bloomfield J, Polman R, O’Donoghue P (2007). Physical demands of different positions in FA Premier League Soccer. J Sports Sci Med..

[16] Carling C, Le Gall F, Dupont G (2012). Analysis of repeated high-intensity running performance in professional soccer. J Sports Sci..

[17] Russell M, Sparkes W, Northeast J (2016). Relationships between match activities and peak power output and creatine kinase responses to professional reserve team soccer match-play. Hum Mov Sci..

[18] Russell M, Northeast J, Atkinson G (2015). Between-match variability of peak power output and creatine kinase responses to soccer match-play. J Strength Cond Res..

[19] Nédélec M, McCall A, Carling C (2013). Physical performance and subjective ratings after a soccer-specific exercise simulation: comparison of natural grass versus artificial turf. J Sports Sci Med..

[20] Buckley JD, Thomson RL, Coates AM (2010). Supplementation with a whey protein hydrolysate enhances recovery of muscle force-generating capacity following eccentric exercise. J Sci Med Sport..

[21] Nosaka K, Sacco P, Mawatari K (2006). Effects of amino acid supplementation on muscle soreness and damage. Int J Sport Nutr Exerc Metab..

[22] White JP, Wilson JM, Austin KG (2008). Effect of carbohydrate-protein supplement timing on acute exercise-induced muscle damage. J Int Soc Sports Nutr..

[23] Rahbek SK, Farup J, de Paoli F (2015). No differential effects of divergent isocaloric supplements on signaling for muscle protein turnover during recovery from muscle-damaging eccentric exercise. Amino Acids..

[24] Shimomura Y, Yamamoto Y, Bajotto G (2006). Nutraceutical effects of branched-chain amino acids on skeletal muscle. J Nutr..

[25] Jackman SR, Witard OC, Jeukendrup AE (2010). Branched-chain amino acid ingestion can ameliorate soreness from eccentric exercise. Med Sci Sports Exerc..

[26] Howatson G, Hoad M, Goodall S (2012). Exercise-induced muscle damage is reduced in resistance-trained males by branched chain amino acids: a randomized, double-blind, placebo controlled study. J Int Soc Sports Nutr..

[27] Cockburn E, Hayes PR, French DN (2008). Acute milk-based protein-CHO supplementation attenuates exercise-induced muscle damage. Appl Physiol Nutr Metab..

[28] Rankin P, Stevenson E, Cockburn E (2015). The effect of milk on the attenuation of exercise-induced muscle damage in males and females. Eur J Appl Physiol..

[29] Cockburn E, Bell PG, Stevenson E (2013). Effect of milk on team sport performance after exercise-induced muscle damage. Med Sci Sports Exerc..

[30] Witard OC, Wardle SL, Macnaughton LS (2016). Protein considerations for optimising skeletal muscle mass in healthy young and older adults. Nutrients..

[31] Tang JE, Moore DR, Kujbida GW (2009). Ingestion of whey hydrolysate, casein, or soy protein isolate: effects on mixed muscle protein synthesis at rest and following resistance exercise in young men. J Appl Physiol..

[32] Gorissen SH, Horstman AM, Franssen R (2016). Ingestion of wheat protein increases in vivo muscle protein synthesis rates in healthy older men in a randomized trial. J Nutr..

[33] Gai Z, Wang Q, Yang C (2016). Structural mechanism for the arginine sensing and regulation of CASTOR1 in the mTORC1 signaling pathway. Cell Discov..

[34] Moore DR, Churchward-Venne TA, Witard O (2015). Protein ingestion to stimulate myofibrillar protein synthesis requires greater relative protein intakes in healthy older versus younger men. J Gerontol A Biol Sci Med..

[35] Macnaughton LS, Wardle SL, Witard OC, et al. The response of muscle protein synthesis following whole-body resistance exercise is greater following 40 g than 20 g of ingested whey protein. Physiol Rep. 2016;4:pii: e12893. doi:10.4814/phy2. 10.14814/phy2.12893 PMC4985555 27511985

[36] Mamerow MM, Mettler JA, English KL (2014). Dietary protein distribution positively influences 24-h muscle protein synthesis in healthy adults. J Nutr..

[37] Glynn EL, Fry CS, Drummond MJ (2010). Muscle protein breakdown has a minor role in the protein anabolic response to essential amino acid and carbohydrate intake following resistance exercise. Am J Physiol Regul Integr Comp Physiol..

[38] Staples AW, Burd NA, West DW (2011). Carbohydrate does not augment exercise-induced protein accretion versus protein alone. Med Sci Sports Exerc..

[39] Koopman R, Beelen M, Stellingwerff T (2008). Coingestion of carbohydrate with protein does not further augment postexercise muscle protein synthesis. Am J Physiol Endocrinol Metab..

[40] McGlory C, Wardle SL, Macnaughton LS, et al. Fish oil supplementation suppresses resistance exercise and feeding-induced increases in anabolic signaling without affecting myofibrillar protein synthesis in young men. Physiol Rep. 2016;4. doi:10.14814/phy2.12715. 10.14814/phy2.12715 PMC4814892 27009278

[41] Parr EB, Camera DM, Areta JL (2014). Alcohol ingestion impairs maximal post-exercise rates of myofibrillar protein synthesis following a single bout of concurrent rraining. PloS One..

[42] Duplanty AA, Budnar RG, Luk HY (2017). Effect of acute alcohol ingestion on resistance exercise induced mTORC1 signaling in human muscle. J Strength Cond Res..

[43] Hong-Brown LQ, Brown CR, Kazi AA (2012). Rag GTPases and AMPK/TSC2/Rheb mediate the differential regulation of mTORC1 signaling in response to alcohol and leucine. Am J Physiol Cell Physiol..

[44] Witard OC, Turner JE, Jackman SR (2014). High dietary protein restores overreaching induced impairments in leukocyte trafficking and reduces the incidence of upper respiratory tract infection in elite cyclists. Brain Behav.

[45] Holway FE, Spriet LL (2011). Sport-specific nutrition: practical strategies for team sports. J Sports Sci..

[46] Balsom PD, Wood K, Olsson P (1999). Carbohydrate intake and multiple sprint sports: with special reference to football (soccer). Int J Sports Med..

[47] Saltin B (1973). Metabolic fundamentals in exercise. Med Sci Sports..

[48] Gunnarsson TP, Bendiksen M, Bischoff R (2013). Effect of whey protein- and carbohydrate-enriched diet on glycogen resynthesis during the first 48 h after a soccer game. Scand J Med Sci Sports..

[49] Krustrup P, Ortenblad N, Nielsen J (2011). Maximal voluntary contraction force, SR function and glycogen resynthesis during the first 72 h after a high-level competitive soccer game. Eur J Appl Physiol..

[50] Hausswirth C, Le Meur Y (2011). Physiological and nutritional aspects of post-exercise recovery: specific recommendations for female athletes. Sports Med..

[51] Burke LM, Collier GR, Hargreaves M (1993). Muscle glycogen storage after prolonged exercise: effect of the glycemic index of carbohydrate feedings. J Appl Physiol..

[52] Fuchs CJ, Gonzalez JT, Beelen M (2016). Sucrose ingestion after exhaustive exercise accelerates liver, but not muscle glycogen repletion compared with glucose ingestion in trained athletes. J Appl Physiol.

[53] Howarth KR, Moreau NA, Phillips SM (2009). Coingestion of protein with carbohydrate during recovery from endurance exercise stimulates skeletal muscle protein synthesis in humans. J Appl Physiol..

[54] Burke LM, Collier GR, Broad EM (2003). Effect of alcohol intake on muscle glycogen storage after prolonged exercise. J Appl Physiol..

[55] Thomas DT, Erdman KA, Burke LM (2016). Position of the Academy of nutrition and dietetics, Dietitians of Canada, and the American College of Sports Medicine: nutrition and athletic performance. J Acad Nutr Diet..

[56] Racinais S, Alonso JM, Coutts AJ (2015). Consensus recommendations on training and competing in the heat. Sports Med..

[57] Shirreffs SM, Sawka MN (2011). Fluid and electrolyte needs for training, competition, and recovery. J Sports Sci..

[58] Sawka MN, Cheuvront S, Kenefick RW (2015). Hypohydration and human performance: impact of environment and physiological mechanisms. Sports Med..

[59] Nose H, Mack GW, Shi XR (1988). Role of osmolality and plasma volume during rehydration in humans. J Appl Physiol..

[60] Takamata A, Mack GW, Gillen CM (1994). Sodium appetite, thrist, and body fluid regulation in humans during rehydration without sodium replacement. Am J Physiol..

[61] Maughan RJ, Leiper JB (1995). Sodium intake and post-exercise rehydration in man. Eur J Appl Physiol Occup Physiol..

[62] Shirreffs SM, Taylor AJ, Leiper JB (1996). Post-exercise rehydration in man: effects of volume consumed and drink sodium content. Med Sci Sports Exerc..

[63] Wemple RD, Morocco TS, Mack GW (1997). Influence of sodium replacement on fluid ingestion following exercise-induced dehydration. Int J Sport Nutr..

[64] Evans GH, Shirreffs SM, Maughan RJ (2009). Postexercise rehydration in man: the effects of osmolality and carbohydrate content of ingested drinks. Nutrition..

[65] Evans GH, Shirreffs SM, Maughan RJ (2011). The effects of repeated ingestion of high and low glucose-electrolyte solutions on gastric emptying and blood 2H2O concentration after an overnight fast. Br J Nutr..

[66] Kamijo Y, Ikegawa S, Okada Y (2012). Enhanced renal Na+ reabsorption by carbohydrate in beverages during restitution from thermal and exercise-induced dehydration in men. Am J Physiol Regul Integr Comp Physiol..

[67] Osterberg KL, Pallardy SE, Johnson RJ (2010). Carbohydrate exerts a mild influence on fluid retention following exercise-induced dehydration. J Appl Physiol..

[68] Desbrow BJ, Jansen S, Barrett A (2014). Comparing the rehydration potential of different milk-based drinks to a carbohydrate-electrolyte beverage. Appl Physiol Nutr Metab.

[69] Watson PL, Love TD, Maughan RJ (2008). A comparison of the effects of milk and a carbohydrate-electrolyte drink on the restoration of fluid balance and exercise capacity in a hot, humid environment. Eur J Appl Physiol.

[70] James LJ, Clayton D, Evans GH (2011). Effect of milk protein addition to a carbohydrate-electrolyte rehydration solution ingested after exercise in the heat. Br J Nutr..

[71] Shirreffs SM, Watson P, Maughan RJ (2007). Milk as an effective post-exercise rehydration drink. Br J Nutr..

[72] Volterman KA, Obeid J, Wilk B (2014). Effect of milk consumption on rehydration in youth following exercise in the heat. Appl Physiol Nutr Metab.

[73] Hobson R, James L (2015). The addition of whey protein to a carbohydrate-electrolyte drink does not influence post-exercise rehydration. J Sport Sci..

[74] James LJ, Gingell R, Evans GH (2012). Whey protein addition to a carbohydrate-electrolyte rehydration solution ingested after exercise in the heat. J Athl Train..

[75] James LJ, Mattin L, Aldiss P (2014). Effect of whey protein isolate on rehydration after exercise. Amino Acids..

[76] Seifert J, Harmon J, DeClercq P (2006). Protein added to a sports drink improves fluid retention. Int J Sport Nutr Exerc Metab..

[77] Calbet JA, Holst JJ (2004). Gastric emptying, gastric secretion and enterogastrone response after administration of milk proteins or their peptide hydrolysates in humans. Eur J Nutr..

[78] Burn-Murdoch RA, Fisher MA, Hunt JN (1978). The slowing of gastric emptying by proteins in test meals. J Physiol..

[79] Baker LB, Jeukendrup AE (2014). Optimal composition of fluid-replacement beverages. Compr Physiol..

[80] Evans GH, James LJ, Shirreffs SM (2017). Optimizing the restoration and maintenance of fluid balance after exercise-induced dehydration. J Appl Physiol (1985).

[81] Sawka MN, Burke LM, Eichner ER (2007). American College of Sports Medicine position stand: Exercise and fluid replacement. Med Sci Sports Exerc..

[82] Kamolrat T, Gray SR (2013). The effect of eicosapentaenoic and docosahexaenoic acid on protein synthesis and breakdown in murine C2C12 myotubes. Biochem Biophys Res Commun..

[83] Smith GI, Atherton P, Reeds DN (2011). Omega-3 polyunsaturated fatty acids augment the muscle protein anabolic response to hyperinsulinaemia-hyperaminoacidaemia in healthy young and middle-aged men and women. Clin Sci (Lond)..

[84] Smith GI, Atherton P, Reeds DN (2011). Dietary omega-3 fatty acid supplementation increases the rate of muscle protein synthesis in older adults: a randomized controlled trial. Am J Clin Nutr..

[85] McGlory C, Galloway SD, Hamilton DL (2014). Temporal changes in human skeletal muscle and blood lipid composition with fish oil supplementation. Prostaglandins Leukot Essent Fatty Acids..

[86] Witard OC, Jackman SR, Breen L (2014). Myofibrillar muscle protein synthesis rates subsequent to a meal in response to increasing doses of whey protein at rest and after resistance exercise. Am J Clin Nutr..

[87] Moore DR, Robinson MJ, Fry JL (2009). Ingested protein dose response of muscle and albumin protein synthesis after resistance exercise in young men. Am J Clin Nutr..

[88] You JS, Park MN, Song W (2010). Dietary fish oil alleviates soleus atrophy during immobilization in association with Akt signaling to p70s6k and E3 ubiquitin ligases in rats. Appl Physiol Nutr Metab..

[89] Albina JE, Gladden P, Walsh WR (1993). Detrimental effects of an omega-3 fatty acid-enriched diet on wound healing. JPEN J Parenter Enteral Nutr..

[90] Calder PC (2012). Mechanisms of action of (n-3) fatty acids. J Nutr..

[91] Jouris KB, McDaniel JL, Weiss EP (2011). The effect of omega-3 fatty acid supplementation on the inflammatory response to eccentric strength exercise. J Sports Sci Med..

[92] Tartibian B, Maleki BH, Abbasi A (2010). The effects of omega-3 supplementation on pulmonary function of young wrestlers during intensive training. J Sci Med Sport..

[93] Gray P, Chappell A, Jenkinson AM (2014). Fish oil supplementation reduces markers of oxidative stress but not muscle soreness after eccentric exercise. Int J Sport Nutr Exerc Metab..

[94] Calder PC (2006). n-3 polyunsaturated fatty acids, inflammation, and inflammatory diseases. Am J Clin Nutr..

[95] Gugus U, Smith C (2010). n-3 fatty acids: a review of current knowledge. Int J Food Sci Tech..

[96] Da Boit M, Gabriel BM, Gray P (2015). The effect of fish oil, vitamin D and protein on URTI incidence in young active people. Int J Sports Med..

[97] National Institutes of Health. Vitamin D fact sheet for health professionals. http://ods.od.nih.gov/factsheets/VitaminD-HealthProfessional/#h8. Accessed Jan 2017.

[98] Heaney R, Garland C, Baggerly C (2015). Letter to Veugelers, P.J. and Ekwaru, J.P., A statistical error in the estimation of the recommended dietary allowance for vitamin D. Nutrients.

[99] Owens DJ, Tang JC, Bradley WJ (2017). Efficacy of high dose vitamin D supplements for elite athletes. Med Sci Sports Exerc..

[100] Owens D, Fraser WD, Close GL (2015). Vitamin D and the athlete: emerging insights. Eur J Sport Sci..

[101] Owens D, Sharples AP, Polydorou I (2015). A systems-based investigation into vitamin D and skeletal muscle repair, regeneration, and hypertrophy. Am J Physiol Endrocrinal Metab..

[102] Stratos I, Li Z, Herlyn P (2013). Vitamin D increases cellular turnover and functionally restores the skeletal muscle after crush injury in rats. Am J Pathol..

[103] Barker T, Henriksen VT, Martins TB (2013). Higher serum 25-hydroxyvitamin D concentrations associate with a faster recovery of skeletal muscle strength after muscular injury. Nutrients..

[104] Ring S, Dannecker EA, Peterson CA (2010). Vitamin D status is not associated with outcomes of experimentally-induced muscle weakness and pain in young, healthy volunteers. J Nutr Metab..

[105] Barker T, Schneider ED, Dixon BM (2013). Supplemental vitamin D enhances the recovery in peak isometric force shortly after intense exercise. Nutr Metab..

[106] Powers SK, Jackson MJ (2008). Exercise-induced oxidative stress: cellular mechanisms and impact on muscle force production. Physiol Rev..

[107] Bouayed J, Bohn T (2010). Exogenous antioxidants: double-edged swords in cellular redox state: health beneficial effects at physiologic doses versus deleterious effects at high doses. Oxid Med Cell Longev..

[108] Pingitore A, Lima GP, Mastorci F (2015). Exercise and oxidative stress: potential effects of antioxidant dietary strategies in sports. Nutrition..

[109] Close GL, Hamilton DL, Philp A (2016). New strategies in sport nutrition to increase exercise performance. Free Radic Biol Med..

[110] Mankowski RT, Anton SD, Buford TW (2015). Dietary antioxidants as modifiers of physiologic adaptations to exercise. Med Sci Sports Exerc..

[111] Jackson MJ (2009). Redox regulation of adaptive responses in skeletal muscle to contractile activity. Free Radic Biol Med..

[112] Gomez-Cabrera MC, Salvador-Pascual A, Cabo H (2015). Redox modulation of mitochondriogenesis in exercise. Does antioxidant supplementation blunt the benefits of exercise training?. Free Radic Biol Med..

[113] Close GL, Ashton T, Cable T (2006). Ascorbic acid supplementation does not attenuate post-exercise muscle soreness following muscle-damaging exercise but may delay the recovery process. Br J Nutr..

[114] Thompson D, Williams C, McGregor SJ (2001). Prolonged vitamin C supplementation and recovery from demanding exercise. Int J Sport Nutr Exerc Metab..

[115] Jakeman P, Maxwell S (1993). Effect of antioxidant vitamin supplementation on muscle function after eccentric exercise. Eur J Appl Physiol Occup Physiol..

[116] Morrison D, Hughes J, Della Gatta PA (2015). Vitamin C and E supplementation prevents some of the cellular adaptations to endurance-training in humans. Free Radic Biol Med..

[117] Paulsen G, Cumming K, Holden G (2014). Vitamin C and E supplementation hampers cellular adaptation to endurance training in humans: a double-blind, randomised, controlled trial. J Physiol..

[118] Bailey DM, Williams C, Betts JA (2011). Oxidative stress, inflammation and recovery of muscle function after damaging exercise: effect of 6-week mixed antioxidant supplementation. Eur J Appl Physiol..

[119] Teixeira VH, Valente H, Casal SI (2009). Antioxidants do not prevent postexercise peroxidation and may delay muscle recovery. Med Sci Sports Exerc..

[120] Braakhuis AJ, Hopkins WG (2015). Impact of dietary antioxidants on sport performance: a review. Sports Med..

[121] Braakhuis AJ (2012). Effect of vitamin C supplements on physical performance. Curr Sports Med Rep..

[122] Sousa M, Teixeira VH, Soares J (2014). Dietary strategies to recover from exercise-induced muscle damage. Int J Food Sci Nutr..

[123] Clements WT, Lee SR, Bloomer RJ (2014). Nitrate ingestion: a review of the health and physical performance effects. Nutrients..

[124] Clifford T, Bell O, West DJ (2016). Antioxidant-rich beetroot juice does not adversely affect acute neuromuscular adaptation following eccentric exercise. J Sports Sci..

[125] Thompson C, Wylie L, Blackwell JR (2017). Influence of dietary nitrate supplementation on physiological and muscle metabolic adaptations to sprint interval training. J Appl Physiol (1985).

[126] Clifford T, Berntzen B, Davison GW (2016). Effects of beetroot juice on recovery of muscle function and performance between bouts of repeated sprint exercise. Nutrients..

[127] Clifford T, Bell O, West DJ (2016). The effects of beetroot juice supplementation on indices of muscle damage following eccentric exercise. Eur J Appl Physiol..

[128] Clifford T, Allerton DM, Brown MA (2017). Minimal muscle damage after a marathon and no influence of beetroot juice on inflammation and recovery. Appl Physiol Nutr Metab..

[129] Bell PG, Stevenson E, Davison GW (2016). The effects of montmorency tart cherry concentrate supplementation on recovery following prolonged, intermittent exercise. Nutrients.

[130] Howatson G, McHugh MP, Hill JA (2010). Influence of tart cherry juice on indices of recovery following marathon running. Scand J Med Sci Sports..

[131] Ammar A, Turki M, Chtourou H (2016). Pomegranate supplementation accelerates recovery of muscle damage and soreness and inflammatory markers after a weightlifting training session. PloS One..

[132] Trombold JR, Reinfeld AS, Casler JR (2011). The effect of pomegranate juice supplementation on strength and soreness after eccentric exercise. J Strength Cond Res..

[133] Hutchison AT, Flieller EB, Dillon KJ (2016). Black currant nectar reduces muscle damage and inflammation following a bout of high-intensity eccentric contractions. J Diet Suppl..

[134] Levers K, Dalton R, Galvan E (2016). Effects of powdered montmorency tart cherry supplementation on acute endurance exercise performance in aerobically trained individuals. J Int Soc Sports Nutr..

[135] Perkins IC, Vine SA, Blacker SD, Willems ME (2015). New Zealand blackcurrant extract improves high-intensity intermittent running. Int J Sport Nutr Exerc Metab..

[136] Cook MD, Myers SD, Blacker SD, Willems ME (2015). New Zealand blackcurrant extract improves cycling performance and fat oxidation in cyclists. Eur J Appl Physiol..

[137] Cook MD, Myers SD, Gault ML (2017). Dose effects of New Zealand blackcurrant on substrate oxidation and physiological responses during prolonged cycling. Eur J Appl Physiol..

[138] Willems ME, Myers SD, Gault ML, Cook MD (2015). Beneficial physiological effects with blackcurrant intake in endurance athletes. Int J Sport Nutr Exerc Metab..

[139] Pialoux V, Mouiner R, Rock E (2009). Effects of acute hypoxic exposure on prooxidant/antioxidant balance in elite endurance athletes. Int J Sports Med..

[140] Walker JB (1979). Creatine: biosynthesis, regulation, and function. Adv Enzymol Relat Areas Mol Biol..

[141] Harris RC, Söderlund K, Hultman E (1992). Elevation of creatine in resting and exercised muscle of normal subjects by creatine supplementation. Clin Sci (Lond)..

[142] Gualano B, Roschel H, Lancha-Jr AH (2012). In sickness and in health: the widespread application of creatine supplementation. Amino Acids..

[143] Rawson ES, Volek JS (2003). Effects of creatine supplementation and resistance training on muscle strength and weightlifting performance. J Strength Cond Res..

[144] Persky AM, Rawson ES (2007). Safety of creatine supplementation. Subcell Biochem..

[145] Jäger R, Purpura M, Shao A (2011). Analysis of the efficacy, safety, and regulatory status of novel forms of creatine. Amino Acids..

[146] Rawson ES, Persky AM (2007). Mechanisms of muscular adaptations to creatine supplementation. Int Sport Med J..

[147] Rawson ES, Clarkson PM, Tarnopolsky MA (2017). Perspectives on exertional rhabdomyolysis. Sports Med..

[148] Greenhaff PL, Bodin K, Soderlund K (1994). Effect of oral creatine supplementation on skeletal muscle phosphocreatine resynthesis. Am J Physiol..

[149] Yquel RJ, Arsac L, Thiaudiere E (2002). Effect of creatine supplementation on phosphocreatine resynthesis, inorganic phosphate accumulation and pH during intermittent maximal exercise. J Sports Sci..

[150] Vandenberghe K, Van Hecke P, Van Leemputte M (1999). Phosphocreatine resynthesis is not affected by creatine loading. Med Sci Sports Exerc..

[151] Volek JS, Rawson ES (2004). Scientific basis and practical aspects of creatine supplementation for athletes. Nutrition..

[152] Nelson AG, Arnall DA, Kokkonen J (2001). Muscle glycogen supercompensation is enhanced by prior creatine supplementation. Med Sci Sports Exerc..

[153] Roberts PA, Fox J, Peirce N (2016). Creatine ingestion augments dietary carbohydrate mediated muscle glycogen supercompensation during the initial 24 h of recovery following prolonged exhaustive exercise in humans. Amino Acids..

[154] Louis M, Poortmans JR, Francaux M (2003). No effect of creatine supplementation on human myofibrillar and sarcoplasmic protein synthesis after resistance exercise. Am J Physiol Endocrinol Metab..

[155] Louis M, Poortmans J, Francaux M (2003). Creatine supplementation has no effect on human muscle protein turnover at rest in the postabsorptive or fed states. Am J Physiol Endocrinol Metab..

[156] Parise G, Mihic S, MacLennan D (2001). Effects of acute creatine monohydrate supplementation on leucine kinetics and mixed-muscle protein synthesis. J Appl Physiol..

[157] Deldicque L, Louis M, Theisen D (2005). Increased IGF mRNA in human skeletal muscle after creatine supplementation. Med Sci Sports Exerc..

[158] Olsen S, Aagaard P, Kadi F (2006). Creatine supplementation augments the increase in satellite cell and myonuclei number in human skeletal muscle induced by strength training. J Physiol..

[159] Willoughby DS, Rosene J (2001). Effects of oral creatine and resistance training on myosin heavy chain expression. Med Sci Sports Exerc..

[160] Willoughby DS, Rosene JM (2003). Effects of oral creatine and resistance training on myogenic regulatory factor expression. Med Sci Sports Exerc..

[161] Safdar A, Yardley NJ, Snow R (2008). Global and targeted gene expression and protein content in skeletal muscle of young men following short-term creatine monohydrate supplementation. Physiol Genomics..

[162] Deminice R, Rosa FT, Pfrimer K (2016). Creatine supplementation increases total body water in soccer players: a deuterium oxide dilution study. Int J Sports Med..

[163] Low SY, Rennie MJ, Taylor PM (1996). Modulation of glycogen synthesis in rat skeletal muscle by changes in cell volume. J Physiol..

[164] Berneis K, Ninnis R, Haussinger D (1999). Effects of hyper- and hypoosmolality on whole body protein and glucose kinetics in humans. Am J Physiol..

[165] Häussinger D, Roth E, Lang F (1993). Cellular hydration state: an important determinant of protein catabolism in health and disease. Lancet..

[166] Cooke MB, Ryballka E, Williams AD (2009). Creatine supplementation enhances muscle force recovery after eccentrically-induced muscle damage in healthy individuals. J Int Soc Sports Nutr..

[167] McKinnon NB, Graham MT, Tiidus PM (2012). Effect of creatine supplementation on muscle damage and repair following eccentrically-induced damage to the elbow flexor muscles. J Sports Sci Med.

[168] Rawson ES, Gunn B, Clarkson PM (2001). The effects of creatine supplementation on exercise-induced muscle damage J Strength Cond Res..

[169] Rosene J, Matthews T, Ryan C (2009). Short and longer-term effects of creatine supplementation on exercise induced muscle damage. J Sports Sci Med Sport..

[170] Machado M, Pereira R, Sampaio-Jorge F (2009). Creatine supplementation: effects on blood creatine kinase activity responses to resistance exercise and creatine kinase activity measurement. Braz J Pharm Sci..

[171] Rawson ES, Conti MP, Miles MP (2007). Creatine supplementation does not reduce muscle damage or enhance recovery from resistance exercise. J Strength Cond Res..

[172] Veggi KF, Machado M, Koch AJ (2013). Oral creatine supplementation augments the repeated bout effect. Int J Sport Nutr Exerc Metab..

[173] Bassit RA, Curi R, Costa Rosa LF (2008). Creatine supplementation reduces plasma levels of pro-inflammatory cytokines and PGE2 after a half-ironman competition. Amino Acids..

[174] Bassit RA, Pinheiro CH, Vitzel KF (2010). Effect of short-term creatine supplementation on markers of skeletal muscle damage after strenuous contractile activity. Eur J Appl Physiol..

[175] Santos RV, Bassit RA, Caperuto EC (2004). The effect of creatine supplementation upon inflammatory and muscle soreness markers after a 30 km race. Life Sci..

[176] Deminice R, Rosa FT, Franco GS (2013). Effects of creatine supplementation on oxidative stress and inflammatory markers after repeated-sprint exercise in humans. Nutrition..

[177] Yuan G, Wahlqvist ML, He G (2006). Natural products and anti-inflammatory activity. Asia Pac J Clin Nutr..

[178] Menon VP, Sudheer AR (2007). Antioxidant and anti-inflammatory properties of curcumin. Adv Exp Med Biol..

[179] Nicol LM, Rowlands DS, Fazakerly R (2015). Curcumin supplementation likely attenuates delayed onset muscle soreness (DOMS). Eur J Appl Physiol..

[180] McFarlin BK, Venable AS, Henning AL (2016). Reduced inflammatory and muscle damage biomarkers following oral supplementation with bioavailable curcumin. BBA Clin..

[181] Sciberras JN, Galloway SD, Fenech A (2015). The effect of turmeric (curcumin) supplementation on cytokine and inflammatory marker responses following 2 hours of endurance cycling. J Int Soc Sports Nutr..

[182] Walker JA, Cerny FJ, Cotter JR (1992). Attenuation of contraction-induced skeletal muscle injury by bromelain. Med Sci Sports Exerc..

[183] Müller S, März R, Schmolz M (2013). Placebo-controlled randomized clinical trial on the immunomodulating activities of low and high-dose bromelain after oral administration: new evidence on the antiinflammatory mode of action of bromelain. Phytother Res..

[184] Vane JR (1971). Inhibition of prostaglandin synthesis as a mechanism of action for aspirin-like drugs. Nat New Biol..

[185] Livio M, Villa S, de Gaetano G (1978). Aspirin, thromboxane, and prostacyclin in rats: a dilemma resolved?. Lancet..

[186] Stone MB, Merrick MA, Ingersoll CD (2002). Preliminary comparison of bromelain and ibuprofen for delayed onset muscle soreness management. Clin J Sport Med..

[187] Shing CM, Chong S, Driller MW (2016). Acute protease supplementation effects on muscle damage and recovery across consecutive days of cycle racing. Eur J Sport Sci..

[188] Buford TW, Cooke MB, Redd LL (2009). Protease supplementation improves muscle function after eccentric exercise. Med Sci Sports Exerc..

[189] Miller PC, Bailey SP, Barnes ME (2004). The effects of protease supplementation on skeletal muscle function and DOMS following downhill running. J Sports Sci Med..

[190] Paxton JZ, Grover LM, Baar K (2010). Engineering an in vitro model of a functional ligament from bone to bone. Tissue Eng Part A..

[191] Shaw G, Lee-Barthel A, Ross ML (2017). Vitamin C-enriched gelatin supplementation before intermittent activity augments collagen synthesis. Am J Clin Nutr..

[192] McAlindon TE, Nuite M, Krishnan N (2011). Change in knee osteoarthritis cartilage detected by delayed gadolinium enhanced magnetic resonance imaging following treatment with collagen hydrolysate: a pilot randomized controlled trial. Osteoarthritis Cartilage..

[193] Clark KL, Sebastianelli W, Flechsenhar KR (2008). 24-Week study on the use of collagen hydrolysate as a dietary supplement in athletes with activity-related joint pain. Curr Med Res Opin..

[194] Oesser S, Adam M, Babel W (1999). Oral administration of (14)C labeled gelatin hydrolysate leads to an accumulation of radioactivity in cartilage of mice (C57/BL). J Nutr..

[195] Heinemeier KM, Olesen JL, Haddad F (2007). Expression of collagen and related growth factors in rat tendon and skeletal muscle in response to specific contraction types. J Physiol..

[196] Nicholson AN, Pascoe PA, Spencer MB (1993). Jet lag and motion sickness. Br Med Bull..

[197] Reilly T, Atkinson G, Waterhouse J (1997). Biological rhythms and exercise.

[198] Hirao A, Tahara Y, Kimura I (2009). A balanced diet is necessary for proper entrainment signals of the mouse liver clock. PloS One..

[199] Oike H, Nagai K, Fukushima T (2011). Feeding cues and injected nutrients induce acute expression of multiple clock genes in the mouse liver. PloS One..

[200] Itokawa M, Hirao A, Nagahama H (2013). Time-restricted feeding of rapidly digested starches causes stronger entrainment of the liver clock in PER2::LUCIFERASE knock-in mice. Nutr Res..

[201] Furutani A, Ikeda Y, Itokawa M (2015). Fish oil accelerates diet-induced entrainment of the mouse peripheral clock via GPR120. PloS One..

[202] Potter GD, Cade JE, Grant PJ (2016). Nutrition and the circadian system. Br J Nutr..

[203] Johnston JD (2014). Physiological links between circadian rhythms, metabolism and nutrition. Exp Physiol..

[204] Mendoza J, Pevet P, Challet E (2008). High-fat feeding alters the clock synchronization to light. J Physiol..

[205] Brager AJ, Ruby CL, Prosser RA (2010). Chronic ethanol disrupts circadian photic entrainment and daily locomotor activity in the mouse. Alcohol Clin Exp Res..

[206] Kräuchi K, Cajochen C, Werth E (2002). Alteration of internal circadian phase relationships after morning versus evening carbohydrate-rich meals in humans. J Biol Rhythms..

[207] Reynolds NC, Montgomery R (2002). Using the Argonne diet in jet lag prevention: deployment of troops across nine time zones. Mil Med..

[208] Forbes-Robertson S, Dudley E, Vadgama P (2012). Circadian disruption and remedial interventions: effects and interventions for jet lag for athletic peak performance. Sports Med..

[209] Boggess BR (2007). Gastrointestinal infections in the traveling athlete. Curr Sports Med Rep..

[210] Stellingwerff T, Pyne DB, Burke LM (2014). Nutrition considerations in special environments for aquatic sports. Int J Sport Nutr Exerc Metab..

[211] Shirreffs SM, Maughan RJ (1997). Restoration of fluid balance after exercise-induced dehydration: effects of alcohol consumption. J Appl Physiol..

[212] WADA. 2017 prohibited list. 2016. http://www.wada-ama.org/en/resources/science-medicine/prohibited-list-documents. Accessed 27 Apr 2017.

